# The phylogenetic analysis of VP1 genomic region in foot-and-mouth disease virus serotype O isolates in Sri Lanka reveals the existence of 'Srl-97', a newly named endemic lineage

**DOI:** 10.1371/journal.pone.0194077

**Published:** 2018-03-23

**Authors:** S. A. E. Abeyratne, S. S. C. Amarasekera, L. T. Ranaweera, T. B. Salpadoru, S. M. N. K. Thilakarathne, N. J. Knowles, J. Wadsworth, S. Puvanendiran, H. Kothalawala, B. K. Jayathilake, H. A. Wijithasiri, M. M. P. S. K. Chandrasena, S. D. S. S. Sooriyapathirana

**Affiliations:** 1 Animal Virus Laboratory, Veterinary Research Institute, Polgolla, Kandy, Sri Lanka; 2 Postgraduate Institute of Science, University of Peradeniya, Peradeniya, Sri Lanka; 3 Department of Molecular Biology and Biotechnology, Faculty of Science, University of Peradeniya, Peradeniya, Sri Lanka; 4 The Pirbright Institute, Pirbright, Woking, Surrey, United Kingdom; Oklahoma State University, UNITED STATES

## Abstract

Foot and mouth disease (FMD) has devastated the cattle industry in Sri Lanka many times in the past. Despite its seriousness, limited attempts have been made to understand the disease to ameliorate its effects–current recommendation for vaccines being based solely on immunological assessments rather than on molecular identification. The general belief is that the cattle population in Sri Lanka acquired the FMD virus (FMDV) strains via introductions from India. However, there could be endemic FMDV lineages circulating in Sri Lanka. To infer the phylogenetic relationships of the FMDV strains in the island, we sequenced the VP1 genomic region of the virus isolates collected during the 2014 outbreak together with a few reported cases in 2012 and 1997 and compared them to VP1 sequences from South Asia. The FMDV strains collected in the 2014 outbreak belonged to the lineage, Ind-2001d, of the topotype, ME-SA. The strains collected in 2012 and 1997 belonged to another lineage called 'unnamed' by the World Reference Laboratory for Foot and Mouth Disease (WRLFMD). Based on the present analysis, we designate the lineage 'unnamed' as Srl-97 which we found endemic to Sri Lanka. The evolutionary rates of Srl-97 and Ind-2001d in Sri Lanka were estimated to be 0.0004 and 0.0046 substitutions/site/year, respectively, suggesting that Srl-97 evolves slowly.

## Introduction

Foot and mouth disease (FMD) is one of the most critical illnesses affecting cattle. The FMD virus (FMDV; family Picornaviridae; genus *Aphthovirus*) causes FMD [1, 2]. The sporadic and countrywide FMD outbreaks have been reported in many parts of the world predominantly in developing regions such as India and many others including Sri Lanka [2, 3, 4, 5, 6, 7, 8]. Seven immunologically distinct serotypes with a genetic diversity of 30–50% in the 1D region (codes for VP1, a capsid protein) of the FMDV genome are available in the literature [9, 10]. The serotype O is the most prevalent strain of FMDV in Pool-2, out of five Pools geographically defined for FMD. The serotype O is the most virulent form that significantly reduces the profit margin of the cattle industry [11]. An FMDV serotype further divides into topotypes which are defined as genetically and geographically distinct genotypes. The critical threshold for topotype differentiation is 15% nucleotide divergence (ND) in the VP1 genomic region [10]. The serotype O has 11 topotypes including Middle East-South Asia (ME-SA), which is the most widespread topotype in South Asia [3, 8, 9, 12]. A topotype further divides into lineages at the critical threshold of 5% ND [10].

Ind-2001 and PanAsia-2, two of the ME-SA lineages, are frequently reported in India [3]. The FMDV was believed to be introduced into Sri Lanka from India through illegal cattle movements [13]. However, no one has ever confirmed this hypothesis. FMD was first reported in Sri Lanka in the 19^th^ century with subsequent outbreaks every four to six years [14, 15]. FMDV samples collected from infected animals in 1950 were confirmed as belonging to serotype O [14, 16]. Two samples collected in 1954 were identified as serotype C by Pirbright, UK [14, 16]. Later, a study confirmed that the causative viral strain for the isolates collected from 1962 to 1967 as serotype O. After three years, the serotype C caused an outbreak which was prevalent from 1970–1981 [14, 16]. Later, a buffalo sample collected in 2009 was found to be serotype O positive [17]. After 2009 no in-depth studies on Sri Lankan FMDV have been published except for a few field surveys and characterizations by World Reference Laboratory for FMD (WRLFMD) reports [18, 19]. A massive FMD outbreak occurred in Jaffna in December 2013, and quickly spread into lower northern regions and subsequently to the other parts of the country by 2014 [20]. Department of Animal Production and Health (DAPH) of Sri Lanka reported 58,645 infected cattle and 1,265 deaths in 2014 [20]. The serotype O caused the 2014 outbreak as confirmed by the WRLFMD [13].

Some viral isolates collected during 2014 were found to be resistant to a specific vaccine produced to control the outbreak. The natural evolution of the virus genome might have contributed to the development of resistance. This highlights the importance of establishing a molecular characterizing scheme in Sri Lanka to identify the exact FMDV lineage for vaccination [9]. Molecular characterization of FMDV lineages is well established in developed countries to facilitate vaccine production [21]. The whole genome sequences of FMDV isolates have recently been utilized to trace the origins of worldwide outbreaks [22, 23, 24]. However, the VP1 genomic region of the FMDV genome is a more practically useful region for molecular diversity analyses to determine the lineages and topotypes [2, 25, 26]. According to WRLFMD reports, three lineages within the topotype ME-SA, have been identified in Sri Lanka. They are Ind-2001, PanAsia-2 and a lineage called ‘unnamed’ [3, 18, 19]. However, no comprehensive phylogenetic analysis on FMDV isolates has been conducted in Sri Lanka to characterize the lineages prevailing in the country [17]. Here we aimed to conduct a phylogenetic analysis using the VP1 sequence data of the isolates collected from 1999–2014 in Sri Lanka. Thereby we idenitified the existence of an endemic FMDV lineage and introduced lineages in Sri Lanka.

## Materials and methods

### Field sampling of FMDV

A major FMD outbreak began in December 2013, from the northern tip of Sri Lanka [13]. The epidemic spread rapidly into many Veterinary Surgeon (VS) Ranges in the country and Veterinary Research Institute (VRI) of DAPH started investigations in January 2014. Initially, the outbreak moved into lower northern regions of the country and subsequently to the other provinces. Approximately, one in twenty infected cattle were reported dead by October 2014 [13]. Out of the many cases diagnosed, 215 clinically-confirmed cases were reported to the VRI by nationwide VS ranges and District-based Veterinary Investigation Centers (VIC) of DAPH. Because of the adverse nature of the epidemic, the DAPH took a policy decision to restrict the collection and movement of samples within the country [27]. Under this restriction, VS ranges and VICs collected and provided only a total of clinically confirmed 67 infected epithelial tissue samples to the VRI. These samples were obtained from ruptured FMD vesicles of clinically diagnosed cattle using the standard procedure described in Hettiarachchi *et al*., (2009) [17].

### RT-PCR and VP1 sequencing

In Sri Lanka, we sequenced the VP1 locus of the FMDV isolated from 10 tissue samples from the 2014 outbreak and two samples from the reported cases in 2012. Another set of 41 tissue samples collected during 1999–2014 outbreak were also sequenced at WRLFMD making a total of 53 sequences for the analysis. Total RNA was extracted from these 53 tissue samples using Qiagen QIAamp viral RNA Minikit (Qiagen, Hilden, Germany). RT-PCR was carried out using Qiagen one-step RT-PCR kit (Qiagen, Hilden, Germany) using *1C-RODI* (5’-TGTTGAAAACTACGGTGGTGA-3’) as the forward primer and *NK72* (5’-GAAGGGCCCAGGGTTGGACTC-3’) as the reverse primer to amplify the VP1 genomic region of the FMDV genome [28]. The PCR products were purified using Promega gel and PCR clean-up system purification kit (Promega, Madison, USA). The purified PCR products were subjected to triplicate DNA sequencing using ABI 3730 DNA Analyzer according to the protocol described in Knowles *et al*., 2016 [29]. We submitted the 53 VP1 sequences generated in this study to GenBank under the accession numbers; MF947453—MF947493 and MF768987—MF768998.

### Sequence alignment and phylogenetic analysis

We constructed an alignment of 639 base pairs in VP1 sequences (S1 Table) of FMDV O/ME-SA in MEGA v 7.0 [30]. The present analysis included 160 VP1 sequences of topotype ME-SA from prevailing lineages in South Asia. The nucleotide substitution model that fits this dataset was inferred using J model test 2.02 [31] in the CIPRES web portal [32]. We implemented Akaike Information Criterion (AIC) to evaluate the robustness of the models [33] and the parameters of the best fitting model, TIM2+I+G [34], to construct phylogenetic trees in further analyses. The phylogenetic relationships within this dataset were inferred in Bayesian framework using BEAST v 2.0 software package [35] in the CIPRES web portal [32]. Since this dataset contains different lineages with unequal diversification rates, we used the Yule model [36] as stochastic branching model in the tree-prior. We implemented two hot and cold chains of Metropolis-coupled Markov Chain Monte Carlo (MCMC) in BEAST v 2.0 [35] for 20 million generations. Our MCMC chains sampled the trees in every 1000 generations while discarding10% of trees as burn-in. To check the performances of MCMC and the posterior distribution of the tree space, we analyzed the log file by using Tracer [37]. Then we constructed the maximum clade credibility tree in TreeAnnotator. In the CIPRES web portal [32], we conducted a bootstrap analysis for 1000 replicates to calculate node support of the phylogenetic tree in RAxML [38] using rapid bootstrap algorithm [39]. Then the robustness of the phenogram was inferred using both bootstrap values and posterior probabilities to gain more accuracy. An Unweighted Pair Group Method with Arithmetic Mean (UPGMA) dendrogram was constructed using uncorrected pairwise distances of all the Sri Lankan FMDV sequences to measure the nucleotide divergence (ND) among lineages circulating in Sri Lanka.

## Results

### Phylogenetic relationships among global and Sri Lankan FMDV strains

**Fig 1:**
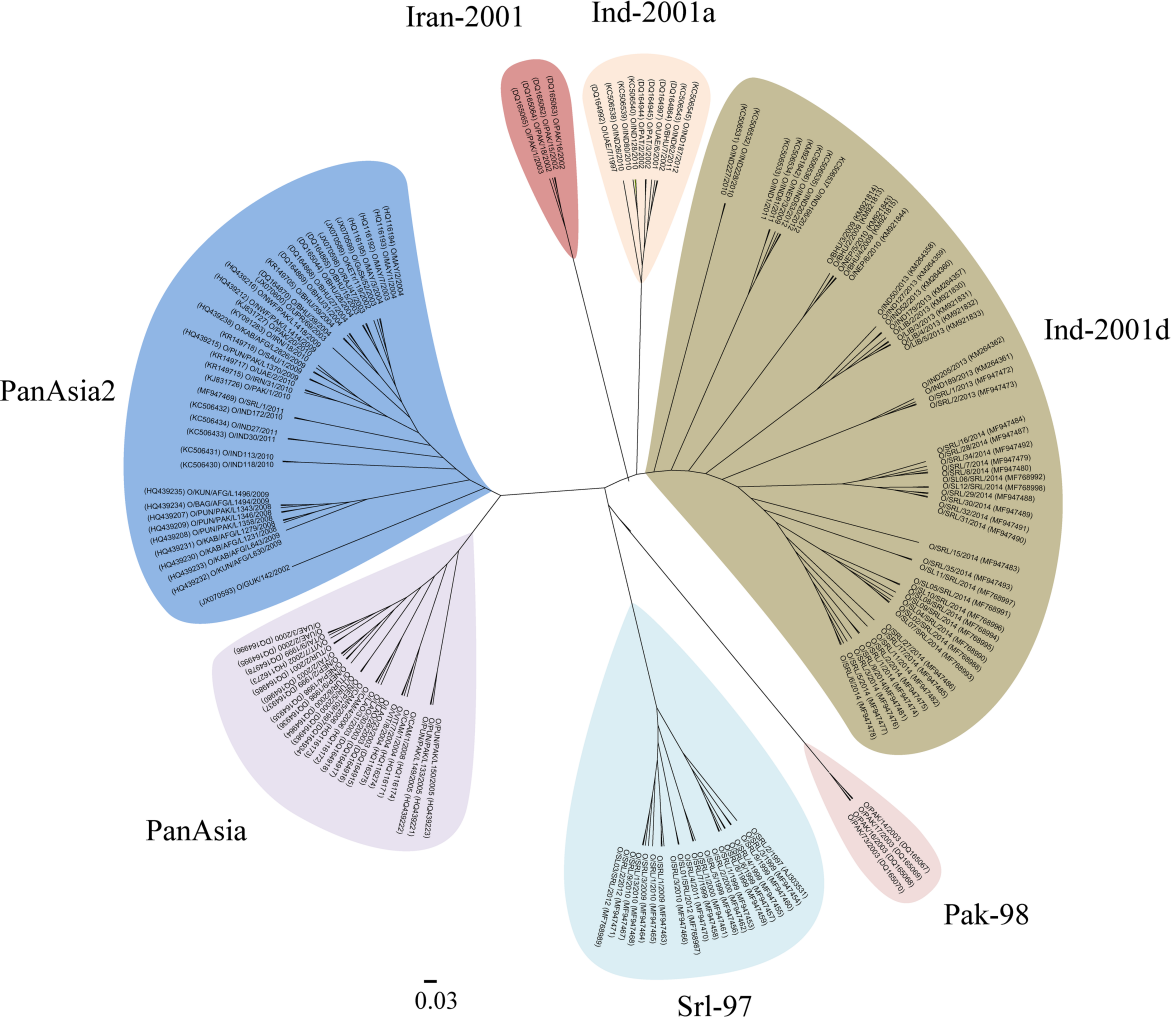
The phylogenetic relationships among the FMDV O/ME-SA strains represented as an unrooted Bayesian maximum clade credibility tree. Srl-97—previously referred to as ‘unnamed’ by WRLFMD.

The tree search using ML and Bayesian criteria produced almost congruent trees with the branches having approximately similar topologies. The higher node support values have indicated the existence of primary clades. The higher posterior probabilities (PP) stabilized the nodes with low bootstrap (bs) values and vice versa (S1 Fig). All the MCMC chains run in our analyses were checked for ESS ≥ 200 to achieve maximum chain convergence. The Bayesian unrooted tree resolved lineages and sub-lineages (Fig 1). Iran-2001, formed a separate branch while sharing monophyly with Ind-2001, whereas Pak-98 positioned as a sister clade with Srl-97 (previously 'unnamed'). Most of the FMDV collected in 2012 and before grouped into the clade, Srl-97, which showed a significant divergence from the lineages PanAsia and Ind-2001. The clade, Srl-97, only contained FMDV collected from Sri Lanka implying the prevalence of an endemic lineage.

### Existence of an endemic lineage in Sri Lanka

**Fig 2:**
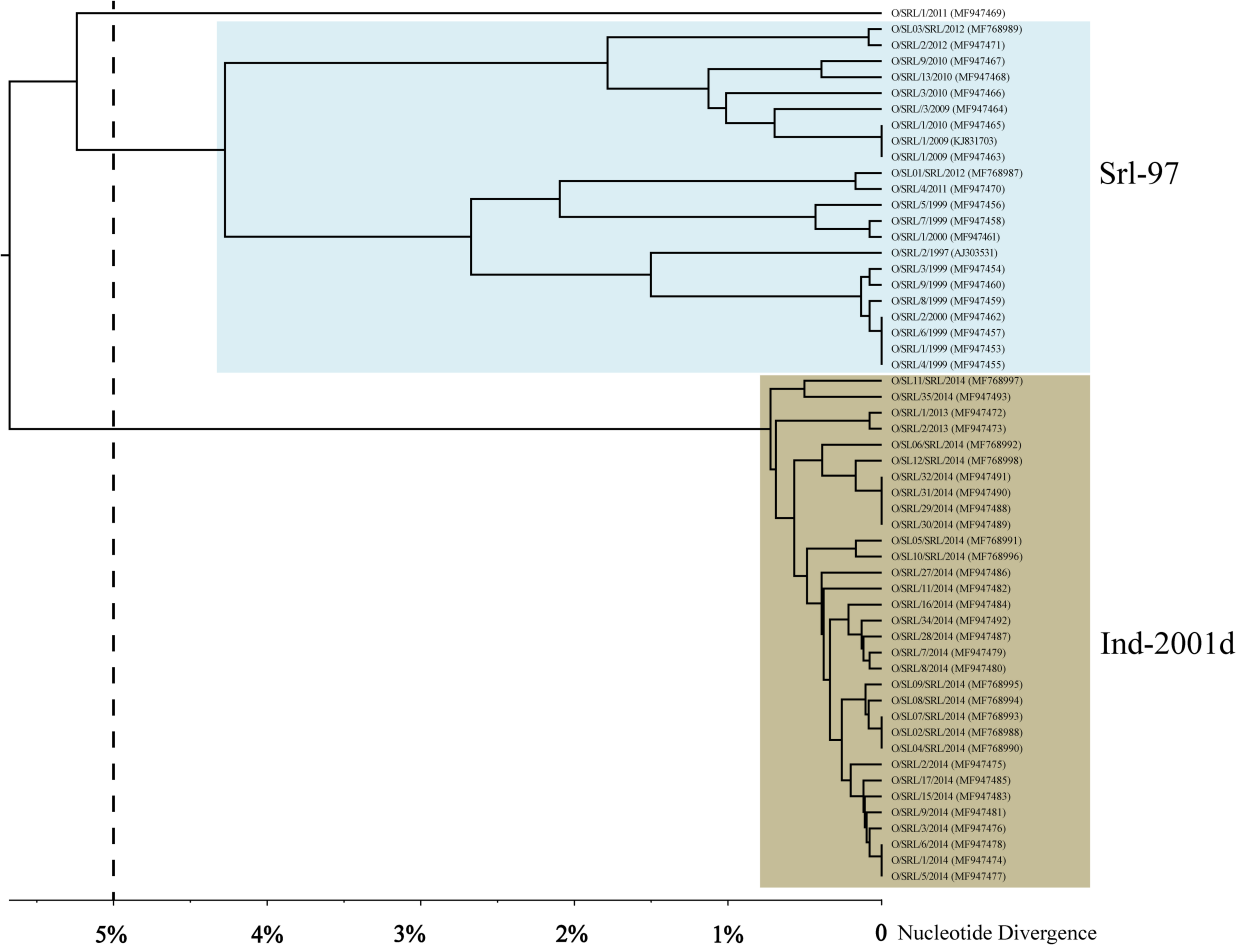
The UPGMA dendrogram showing the nucleotide divergences (ND) among FMDV ME-SA isolates in Sri Lanka. The X-axis shows the percentage nucleotide divergence (% ND). The colored boxes indicate different lineages. The lineages showed here tally with the clades in Bayesian MCC tree (Fig 2). The WRLFMD previously labeled Srl-97 as 'unnamed'.

All the FMDV isolates from Sri Lanka separated into three clades in the distance tree with an ND of 5% indicating the presence of three separate lineages (Fig 2). Ind-2001d separated with an ND of 5.68% from the rest and PanAsia2, separated from Srl-97 with an ND of 5.24%. Srl-97 contained two clades showing an ND of 4.27%. The nucleotide substitution rates calculated for Ind-2001d and Srl-97 were 4.6 × 10^−3^ substitutions/site/year and 4 × 10^−4^ substitutions/site/year, respectively, revealing the existence of an independently evolving lineage within the country.

## Discussion

FMD is the most damaging viral disease affecting cattle farming in Sri Lanka. Thus, the Sri Lankan government has prioritized FMD, among all other livestock diseases, to be eradicated by 2020 [20]. Despite the severity of the disease, only a limited number of attempts have been made in Sri Lanka to track the origins of outbreaks and to determine the lineage and topotype of circulating viral strains. Although FMD is prevalent in the country since 1869 with occurrences of sporadic outbreaks from time to time, no studies have determined the evolution of the FMDV through a complete analysis. The interpretation of the phylogenetic relationships among the FMDV strains in Sri Lanka was not possible due to the unavailability of an adequate number of viral sequences. The lack of FMDV molecular characterization attempts caused the inability to establish a precise molecular characterization scheme which, in return, hinders the proper control measures against the disease. Therefore, the present study deemed it necessary to fill the existing knowledge gap of characterization, evolution, and origins of FMDV strains prevailing in the country and the information generated would be critical for the development and deployment of effective vaccines in the future.

In the present phylogenetic analysis, the unrooted MCC tree resulted in eight distinct clusters. They represent the major lineages and sub-lineages within the topotype ME-SA. Our study separates FMDV isolates of Sri Lanka into two clades. The first clade includes the isolates collected during the outbreak in 2014 belonging to Ind-2001d sub-lineage. The second clade, previously labeled as 'unnamed' [3, 18, 19], includes the FMDV isolates and the sequences collected from 1997 to 2012. The stem of the second clade connects with Pak-98. Thus the second clade is phylogenetically more closely related to Pak-98 than to Ind-2001. The higher bootstrap and posterior probability values strongly supported the existence of the second clade (S1 Fig). We observed a single FMDV isolate of PanAsia2 in 2011; however, we cannot explain the evolutionary aspects of the PanAsia2 in Sri Lanka based on just one sequence. The classification of FMDV isolates collected from Sri Lanka into three distinctly separated clusters indicates the prevalence of three lineages within the country from 1997 to 2014. However, it is noteworthy, that, the cluster which includes previously reported Sri Lankan sequences classified as 'unnamed' solely comprises Sri Lankan sequences collected from 1997 to 2012, and therefore, we suggest to rename this new lineage as 'Srl-97' which appears to be an endemic lineage prevalent in Sri Lanka.

The three clades observed in the UPGMA tree (Fig 2) exhibit an ND greater than 5% from each other. Thus we can claim that Srl-97 represents a distinct lineage in Sri Lanka. The clade structure displayed in MCC unrooted tree (Fig 1) further verifies the existence of Srl-97. In this study, the estimated rate of evolution for sub-lineage, Ind-2001d, in Sri Lanka (4.6 × 10–3 substitutions/site/year) is in parallel to the reported rates for Ind-2001d in Vietnam [40] and India [41]. This observed rate of evolution for Ind-2001d in Sri Lanka is lower than the rate observed during a defined epizootic outbreak [42]. Sri Lanka is a small island (total area of 65,610 km2) with poorly managed and sparse cattle farms slowing the rate of disease spread compared to that in India. The low livestock density of Sri Lanka (10–20 animals/km^2^) compared to India (100–250 animals/km^2^) may also lower the rate of the evolution of FMDV [8]. The observed evolutionary rate of the lineage, Srl-97 is 4 × 10^−4^ substitutions/site/year which is less than that of the long-term evolutionary rate of the VP1 genomic region for all FMDV. It seems that the linage, Srl-97, evolved independently from the rest to reach an evolutionary-static stage over a period of 15 years. However, the probability of this lineage to reemerge by acquiring the needful number of synonymous mutations, thus giving rise to an outbreak cannot be ruled out. There are reported sudden viral outbreaks from unnoticed stages for long periods of time [41]. In such situations, the relative genetic stability alternates periodically between rapid and static evolutionary stages. The molecular epidemiological inferences made in the present study on the lineage, Srl-97, would be useful to get prepared ahead of the possible further outbreaks of FMD.

## Conclusions

The phylogenetic and evolutionary analyses of FMDV strains prevalent in Sri Lanka provided two key inferences. The sub-lineage ‘Ind-2001d’ with an Indian origin was the etiologic agent of the outbreak reported in 2014. The current study documents, for the first time, the existence of an endemic lineage “Srl-97” in Sri Lanka which was previously referred to as ‘unnamed’ by WRLFMD.

## Supporting information

S1 Table The *VP1* DNA sequences of the FMDV ‘Serotype O’ used in the present study. nk; not known.
(XLSX)

S1 Fig The MCC tree drawn in Bayesian criteria. The node support values are displayed on the particular branches of the cladogram. Bootstrap values (bs) are indicated below the node and posterior probabilities (PP) are indicated above the node. The nodes with PP higher than 85 and bs higher than 95 are only shown.
(TIF)
