# Correction: The phylogenetic analysis of VP1 genomic region in foot-and-mouth disease virus serotype O isolates in Sri Lanka reveals the existence of 'Srl-97', a newly named endemic lineage

**DOI:** 10.1371/journal.pone.0196491

**Published:** 2018-04-23

**Authors:** S. A. E. Abeyratne, S. S. C. Amarasekera, L. T. Ranaweera, T. B. Salpadoru, S. M. N. K. Thilakarathne, N. J. Knowles, J. Wadsworth, S. Puvanendiran, H. Kothalawala, B. K. Jayathilake, H. A. Wijithasiri, M. M. P. S. K. Chandrasena, S. D. S. S. Sooriyapathirana

In the Funding section, the grant number for the funder National Research Council of Sri Lanka; Research Grant Scheme: Target Oriented is listed incorrectly. The correct grant number is: NRC/TO/14-10.
